# Exploring the Nexus Between Emulsifier Characteristics and *Salmonella* Typhimurium Viability in Oil‐in‐Water Emulsions

**DOI:** 10.1002/fsn3.4569

**Published:** 2024-11-04

**Authors:** Shawn Tsai, Rohan V. Tikekar

**Affiliations:** ^1^ Department of Nutrition and Food Science University of Maryland College Park Maryland USA

**Keywords:** emulsifier, emulsion, molecular weight, *Salmonella* Typhimurium, surface charge

## Abstract

Molecular characteristics of emulsifiers such as their molecular weight (MW) and surface charge, not only affect the stability of the emulsion but also can have an impact on its capacity to either inhibit or promote microbial proliferation. These characteristics can affect the behavior of pathogens such as *Salmonella* Typhimurium in emulsion systems. The growth and thermal resistance of *S*. Typhimurium were monitored at different oil content levels (20%, 40%, and 60%) in emulsions stabilized by three whey protein‐based emulsifiers: whey protein isolate (WPI), whey protein hydrolysate (WPH), and a modified WPI with an alteration of charge (WPI^+^). Our study revealed that emulsifier itself with different MW and surface charge had no effect on bacterial growth and inactivation without oil inclusion (*p* > 0.05). However, it was found that higher bacterial growth rate at 60% oil content emulsion stabilized with WPI^+^ (0.65 ± 0.03 log CFU/h) than WPI (0.19 ± 0.04 log CFU/h), which showed the charge of emulsifiers has different effects on microbial dynamics in oil‐in‐water emulsion. Interestingly, WPI^+^ in emulsions also seemed to convey protection against thermal inactivation of bacteria. These data describe a complex interrelationship between the physicochemical characteristics of the emulsifier and its interacting nature with bacterial cells. They throw even more light on the insight about the importance of a strategic approach toward emulsifier selection in food formulations. This is crucial for the food safety and stability of products.

## Graphical abstract

Appropriate emulsifier selection, considering molecular weight (MW) and surface charge, is instrumental in attaining desired product characteristics while controlling bacterial growth and spoilage within emulsion system. However, it is unclear whether MW or surface charge of emulsifier has any effect on bacterial growth and inactivation within emulsion. To test this, oil‐in‐water emulsions were formulated using three distinct emulsifiers: whey protein isolate (WPI), whey protein hydrolysate (WPH), and whey protein isolate under a pH lower than the isoelectric point (WPI^+^), with varying oil proportions (20%, 40%, and 60% [v/v]). *Salmonella* growth and inactivation rates were compared in these emulsions.

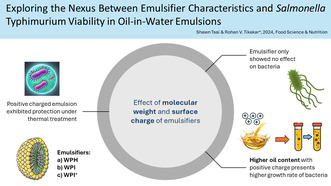

## 1 Introduction

Understanding the dynamics of pathogen behavior within emulsion systems is essential for ensuring food safety, as mishandling can lead to spoilage or contamination of products (Keerthirathne et al. 2016, 2019; Leistner and Gorris 1995). In this respect, identifying key factors that influence both growth and neutralization of microbes in such systems is critical for developing effective food preservation methods (Podolak, Whitman, and Black 2020). In food industry, emulsifiers are widely recognized for their role in enhancing the texture and stability of emulsified products (Tan and McClements 2021). The choice of emulsifier, influenced by its molecular weight (MW), significantly affects the overall sensory and physical qualities of products. Low MW emulsifiers, like mono‐ and diglycerides, are favored for their ability to improve transparency and flavor profile of foodstuffs, while their high MW counterparts, including specific protein and polysaccharide types, are valued for their capacity to maintain product integrity by preventing oil droplet aggregation (Moonen and Bas 2014; Nooshkam et al. 2023). However, the literature seldom addresses how the MW of emulsifiers directly impacts the proliferation or reduction of bacterial populations in emulsion‐based foods.

Emulsifiers are classified based on their surface charge into three main types: neutral, anionic (which have a negative charge), and cationic (which carry a positive charge) (Marhamati, Ranjbar, and Rezaie 2021). The interaction between the charge of emulsifier and bacterial cells, which is influenced by electrostatic forces, is crucial in shaping the microbial environment within emulsions (Ly et al. 2008). Anionic emulsifiers tend to repel bacteria bearing similar negative charges, potentially mitigating unwanted microbial proliferation on food interfaces (Wang et al. 2023). Conversely, neutral emulsifiers do not contribute to electrostatic interactions with bacterial cells, hence may not influence bacterial growth within the emulsion (de Toledo et al. 2021). Positively charged emulsifiers, on the other hand, may exhibit affinity toward bacterial surfaces, possibly agglomerating to obstruct nutrient ingress or thermal energy transfer into bacterial cells during thermal treatment (Satpute et al. 2010). Therefore, selecting emulsifiers with the right balance of characteristics is crucial not only for achieving specific product qualities but also for minimizing the risk of microbial spoilage. A comprehensive body of literature has explored the efficacy of emulsifiers in decreasing bacterial growth across a variety of food systems, spanning fresh produce, biofilms, and dairy products, exhibiting substantial reductions in various pathogen populations (Kang and Song 2015; Kolomaznik, Nova, and Calkovska 2017; Simões, Pereira, and Vieira 2005). Building on these insights, our study aimed to the nuanced interrelations between emulsifier MW and surface charge and their collective impact on the growth and thermal inactivation of bacteria, an area that remains underexplored in the current body of knowledge.

This study focused on investigating contributing factors influencing both the growth rate of *S*. Typhimurium and their inactivation rate in oil‐in‐water emulsions. Vegetable oil was selected as a model substrate and employed three variants of the same base emulsifier, whey protein, to maintain consistent chemical composition while varying MW and surface charge. These variants included: (1) WPI, representing an anionic emulsifier; (2) WPH, which is a hydrolyzed form of WPI with reduced MW; and (3) WPI^+^, where WPI was adjusted to a pH below its isoelectric point (5.5), altering its surface charge without affecting its primary chemical structure (Durham and Hourigan 2007). This methodology enabled controlled, systematic variation of emulsifier properties, facilitating assessment of their impact on microbial dynamics within emulsion systems.

## 2 Materials and Methods

### 2.1 Materials

The *Salmonella enterica* serotype Typhimurium strain (CVM98) used in this study originated from the microbial collection maintained in the Department of Nutrition and Food Science, University of Maryland, College Park. The tryptic soy agar (TSA) and tryptic soy broth (TSB), the growth media used in this project were obtained from BD Biosciences in Franklin Lakes, NJ, and were identified by their product numbers as TSA (236920) and TSB (211825), respectively. Buffered peptone water (BPW) used for the oxygen uptake experiments was obtained from Thermo Scientific Remel Agar, Waltham, MA, with the following catalog number, R452672. The emulsifiers used in this study include whey protein isolate (WPI), with MW approximated to 18,000 Da, and whey protein hydrolysate (WPH), with MW between 450 and 500 Da, both obtained from Hilmar Ingredients, Hilmar, CA, product codes Hilmar 9400 and Hilmar 8390, respectively, for WPI and WPH. Soybean oil was the major vegetable oil used in this study; and it was obtained locally and stored in dark brown bottles at room temperature.

### 2.2 Methods

#### 2.2.1 Sample Preparation

In the experimental setup, each TSB medium was supplemented with a 2.0% (v/v) concentration of either WPI or WPH. Different quantities of this solution, including 10, 8, 6, and 4 mL, were blended and then homogenized to achieve a homogenous mixture. Then, to create oil‐in‐water emulsions with 0%–60% (v/v) oil concentrations, specific quantities of vegetable oil were mixed into the TSB‐emulsifier solution, with the total volume brought up to 10 mL. This blend underwent ultrasonic homogenization at 200 W for 3 min using an ultrasonic processor (model FB505; Fisher Scientific, Hampton, NH). In the case of emulsions with WPI at a positive charge (WPI^+^), the pH adjustment to 5.0, below the isoelectric point (PI) of 5.5, was strategic. These adjustments instigated a positive surface charge, thereby transforming its charge attributes, as documented by Durham and Hourigan (2007).

#### 2.2.2 Physical and Chemical Properties

To assess the emulsifier solutions and emulsions without *S*. Typhimurium, we prepared 10 mL samples for detailed evaluation. pH levels were measured with a Fisher Scientific pH meter (model AB15; Hampton, NH), and water activity was checked using a HygroPalm device (model HP23‐A, Rotronic, Hauppauge, NY). For determining particle size and zeta (ζ) potential, we combined a Particle Sizer with a ζ‐Potential Analyzer (NanoBrook Omni, Brookhaven Instruments, Holtsville, NY). This combination was achieved by modifying the cube and adjusting the setup settings in the software, allowing for seamless integration and simultaneous measurement of both parameters. Emulsion samples were diluted in distilled water at a 1:10,000 ratio before size analysis. We employed dynamic light scattering (DLS) for particle size and distribution, and electrophoretic light scattering (ELS) for ζ‐potential, using a 10 mW Helium–Neon (HeNe) laser at 633 nm wavelength to measure emulsion particle diameters. To ensure accuracy, all DLS and ELS measurements were conducted in triplicate at a steady temperature of 25°C.

#### 2.2.3 Bacterial Growth and Enumeration

Frozen *S*. Typhimurium was initially thawed and plated onto TSA plates in an overnight incubation at 37°C, extending for about 20 h. After the initial incubation, the plates were stored at 4°C to survive up to a month. For subsequent experiments, *S*. Typhimurium was selected from these TSA plates. The bacterial suspensions were then diluted to achieve 5 log CFU/mL. Suspensions were then added to various media including TSB, emulsifier solutions, and emulsions. The prepared samples were incubated at 37°C for specific time periods (0, 2, 4, 6, 8, 12, 24 h). At each point, samples were evenly spread on TSA plates using a spiral platter (Eddy Jet; Neutec Group Inc., Farmingdale, NY). The plates were then incubated for about 20 h at 37°C. Finally, the number of bacterial colonies was determined using the IUL Flash & Go system (6010 colony counter; Neutec Group Inc.).

#### 2.2.4 Thermal Treatment

This study focused on the thermal inactivation of *S*. Typhimurium in different emulsion setups by first incorporating the bacteria into TSB, various emulsifier solutions, and emulsions. Following a stabilization period of around 20 h at 37°C, 200 μL samples from each group were subjected to a temperature of 55°C in a water bath (Model: 28 L‐M; Fisher Scientific) to assess thermal inactivation, a temperature proven by previous studies (Jin et al. 2009; Park and Kang 2013; Sung et al. 2014). At defined time points (every 15 min up to 90 min), samples were retrieved, spread onto TSA plates, and incubated for around 20 h at 37°C. Colony counts were acquired using plate counter, facilitating the evaluation of bacterial resistance to elevated temperature exposure.

#### 2.2.5 Statistical Analysis

To ensure the validity of our experiment, we replicated each procedure three times. We analyzed bacterial growth patterns using a logistic growth model, described in Equation (1) as:

(1) $$\log N_{t}=\log N_{0}+\frac{C}{1+e^{-\mu \left(t-d\right)}}$$

In this equation, *N*_*t*_ represents the bacterial count at any given time *t*, *N*_0_ is the initial bacterial count, *C* is the maximum logarithmic increase in bacterial count from the initial phase to the peak, *μ* is the maximum growth rate, *d* is the time at the curve's inflection point. We determined the lag phase duration using by Buchanan and Cygnarowicz (1990), shown in Equation (2):

(2) $$\lambda =d-\left(\frac{1}{\mu }\right)$$

where *λ* denotes the lag phase duration. For the evaluation of bacterial reduction at 55°C, we employed the Weibull model, as shown in Equation (3):

(3) $$\log N_{t}=\left(\frac{-1}{2.303}\right){\left(\frac{t}{\alpha }\right)}^{\beta }$$

Here, *N*_*t*_ again denotes the bacterial count at time *t*, with *α* and *β* being the Weibull model's shape and scale parameters, respectively, as interpreted by van Boekel (2002). The 5D value, or the time required for a 5‐log reduction in bacterial count, was calculated using Equation (4):

(4) $$5D=\alpha {\left(-\ln\left(10^{-5}\right)\right)}^{\frac{1}{\beta }}$$

Statistical analyses, including multiple *t*‐test and Bonferroni correction, were applied to assess the significant differences between groups by JMP Pro 15.2.0, with a significance level set at *α* = 0.05. To ensure consistency, all experimental trials were performed in triplicate.

## 3 Results and Discussion

### 3.1 Evaluation of Physical and Chemical Properties

The examination of o/w emulsions, including concentrations of 0%, 20%, 40%, and 60% with a 2.0% inclusion of WPI and WPH, yielded insights into their physical and chemical characteristics, as detailed in Table 1. The pH levels for both WPI and WPH emulsifier solutions were normalized to a neutral value of approximately 7.0, with no statistically significant variance observed across different emulsion compositions (*p* > 0.05). To alter the surface charge of WPI to its cationic form (WPI^+^), the pH was reduced to about 5.0, positioning it beneath the PI of WPI. Across all emulsion formulations, water activity was uniformly maintained at an average value of 0.99, indicating minimal variability (Table 1). Notably, the choice of emulsifier influenced the size of emulsion droplets: WPI, WPI^+^, and WPH produced average droplet diameters of approximately 500 nm, 10, and 50 μm, respectively (Table 1). The use of WPH was associated with a notable increase in average droplet size across varying oil content levels, which is consistent with previous observations of WPH limited emulsifying efficiency and its tendency toward larger droplet distributions (van der Ven et al. 2001). Despite the observed variations in droplet size attributed to molecular weight and surface charge differences, the physical stability of the emulsions, in terms of remaining a single phase without separation, remained unaffected over a 24‐h observation period (Data not shown). This finding is supported by earlier research indicating that droplet size, ranging from nano to microscale, does not significantly alter bacterial growth or inactivation rates (Brocklehurst et al. 1995; Buranasuksombat et al. 2011). The ζ‐potential measurements for WPI and WPI^+^ in 20%, 40%, and 60% emulsions revealed values of approximately −10 and +4 mV, respectively (Table 1). This variation in charge is attributed to the pH adjustments relative to the PI of the whey protein, with a negative charge prevailing at pH levels above the PI and a positive charge manifesting below it.

**TABLE 1:** Characteristics of the emulsions, each value represents the mean derived from three separate experiments, with the standard deviation (SD) provided to indicate the variability of the measurements.

Emulsifier	Oil fraction (v/v)	pH	Aw	Particle size (nm)	Zeta potential (mV)
TSB	0%	6.99 ± 0.01	1.00 ± 0.01	—	—
WPI	0%	6.91 ± 0.00	0.99 ± 0.01	—	—
	20%	6.91 ± 0.01	0.99 ± 0.01	448.6 ± 29.5	−8.66 ± 0.41
	40%	6.90 ± 0.04	0.99 ± 0.01	788.8 ± 403.1	−9.83 ± 2.22
	60%	6.85 ± 0.03	0.99 ± 0.01	695.5 ± 26.3	−12.32 ± 0.98
WPH	0%	6.91 ± 0.00	0.99 ± 0.01	—	—
	20%	6.91 ± 0.00	0.99 ± 0.01	9647.5 ± 4484.7	—
	40%	6.90 ± 0.03	0.99 ± 0.01	> 10,000	—
	60%	6.89 ± 0.02	0.99 ± 0.01	> 10,000	—
WPI^+^	0%	5.11 ± 0.02	0.99 ± 0.01	—	—
	20%	5.11 ± 0.01	0.99 ± 0.01	> 10,000	+5.63 ± 0.36
	40%	5.15 ± 0.02	0.98 ± 0.01	4475.4 ± 4806.5	+4.10 ± 0.78
	60%	5.12 ± 0.01	0.98 ± 0.01	> 10,000	+2.77 ± 1.02

### 3.2 Influence of Emulsifier Molecular Characteristics on *S*. Typhimurium Growth Behavior

**FIGURE 1:**
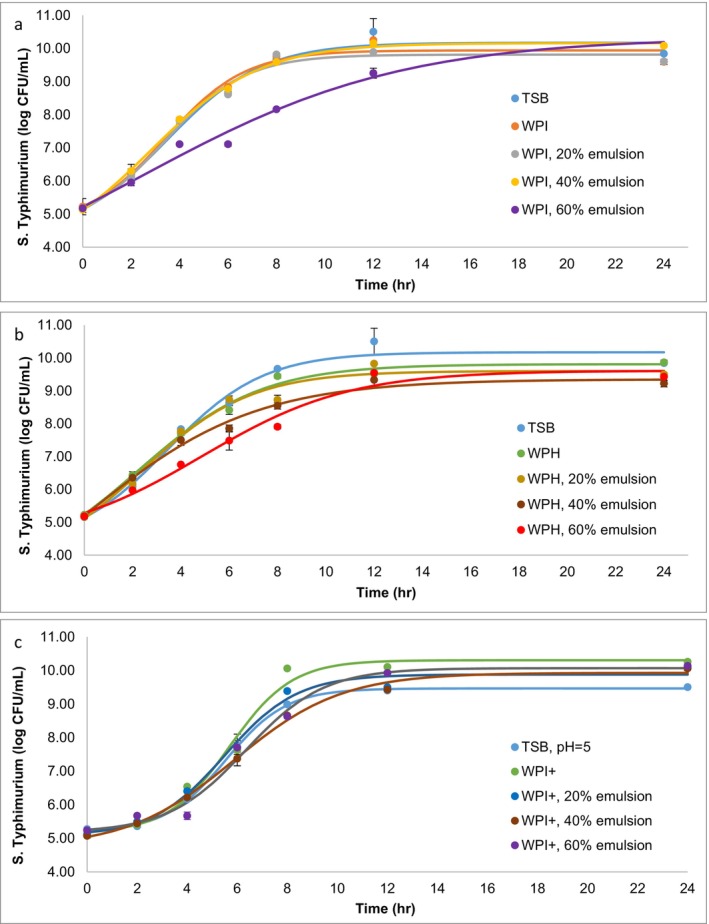
Growth dynamics of *S*. Typhimurium at 37°C across different mediums: (a) TSB and WPI solutions, alongside emulsions with 20%, 40%, and 60% oil content; (b) TSB and WPH solutions, alongside emulsions with 20%, 40%, and 60% oil content; (c) TSB adjusted to pH 5.0, WPI^+^ solutions, and emulsions containing 20%, 40%, and 60% oil content. The logistic growth model was applied to fit the experimental data, with the solid lines representing the average model fit for the observed growth patterns. Each data point reflects the mean of three replicates, presented with the corresponding standard deviation (SD).

The growth models of *S*. Typhimurium in TSB, alongside three distinct emulsifier solutions and their corresponding emulsions, are depicted in Figure 1. Utilizing the logistic growth model, which demonstrated a strong correlation (*R* ^2^ > 0.98), we analyzed critical growth parameters such as the maximum growth rate (log CFU/h) and the lag phase duration (h), with findings summarized in Table 2.

**TABLE 2:** Growth dynamics of *S*. Typhimurium across various mediums, including TSB, different emulsifier solutions, and their corresponding emulsions at 37°C.

	TSB	WPI	WPI, 20% emulsion	WPI, 40% emulsion	WPI, 60% emulsion
*R* ^2^	0.98	0.99	0.99	0.99	0.99
Growth rate	0.51 ± 0.02^c,d^	0.61 ± 0.04^b^	0.60 ± 0.05^b,c^	0.44 ± 0.04^d,e^	0.19 ± 0.04^h^
Lag phase (h)	1.48 ± 0.15^d^	1.75 ± 0.32^d^	1.64 ± 0.43^d^	0.59 ± 0.51^e^	NA

		WPH	WPH, 20% emulsion	WPH, 40% emulsion	WPH, 60% emulsion
*R* ^2^	—	0.99	0.98	0.99	0.98
Growth rate	—	0.39 ± 0.05^e,f^	0.44 ± 0.03^d,e^	0.31 ± 0.02^g^	0.33 ± 0.04^f,g^
Lag phase (h)	—	NA	NA	NA	1.75 ± 1.10^d^

	TSB, pH = 5	WPI^+^	WPI^+^, 20% emulsion	WPI^+^, 40% emulsion	WPI^+^, 60% emulsion
*R* ^2^	0.99	0.98	0.99	0.99	0.99
Growth rate	0.83 ± 0.04^a^	0.83 ± 0.01^a^	0.70 ± 0.03^b^	0.47 ± 0.03^d^	0.65 ± 0.03^b^
Lag phase (h)	4.34 ± 0.19^b^	4.52 ± 0.31^a,b^	4.08 ± 0.14^b,c^	3.74 ± 0.31^c^	4.77 ± 0.06^a^

*Note:* The values presented are the averages from three replicates, each accompanied by the standard deviation (SD) to reflect measurement variability. To elucidate significant distinctions across the groups, alphabetic labels (a, b, c, etc.) were employed, as determined by a series of multiple *t*‐tests. Bonferroni correction was then proceeding to avoid Type I error. Groups sharing a common letter do not exhibit statistically significant differences (*p* > 0.05), whereas distinct letters denote significant disparities (*p* < 0.05). The term “NA” denotes scenarios where lag phase calculations were not applicable. The bacterial growth rates are expressed in units of log CFU/h.

Analysis presented in Table 2 indicated the initial adaptation period of *S*. Typhimurium, represented by lag phase duration, was similar in both WPI solutions and TSB (*p* > 0.05), suggesting a comparable initial bacterial acclimatization in these mediums. However, WPH solutions did not exhibit a distinct lag phase, indicating an immediate onset of bacterial growth. Furthermore, growth rate in WPI solution was higher compared with that in TSB (*p* < 0.05), whereas WPH solution exhibited a significantly reduced bacterial growth rate compared with TSB and WPI solutions (*p* < 0.05). This suggested that emulsifiers with a lower MW might modestly impede *S*. Typhimurium growth. Therefore, lower MW emulsifier (WPH) may influence both growth rate and lag phase duration of *S*. Typhimurium at 37°C. Additional examinations in WPI^+^ solutions showed growth rates and lag phases analogous to those in TSB at a pH of 5.0 (*p* > 0.05), with similar patterns observed in WPI solutions. Hence, the result revealed that the predominant influence of molecular weight over surface charge on bacterial growth in these solutions.

The study further explored how different oil concentrations within the emulsions impacted *S*. Typhimurium growth dynamics. It was found that bacterial growth response to increasing oil content varied based on the type of emulsifier used. In emulsions stabilized with WPI, 60% oil concentration led to a significant reduction in both lag phase duration and growth rate (*p* < 0.05). In contrast, the growth rate in emulsions stabilized with WPH remained consistent regardless of oil content increases, potentially due to the minimal interaction between the smaller protein peptides in WPH and the oil droplets (Schröder et al. 2017). Interestingly, WPI^+^‐stabilized emulsions exhibited significant variations. Emulsions with a 60% oil concentration showed increased growth rates and extended lag phases compared to those with 20% and 40% oil concentration (*p* < 0.05), indicating an oil concentration‐dependent effect on bacterial growth dynamics. This highlights the significant role of oil content in influencing emulsion characteristics according to the type of emulsifier use, a finding not previously documented.

Previous research has indicated that the growth rate and lag phase of *S*. Typhimurium are affected by the acidity level at 37°C (Chung and Goepfert 1970; Theys et al. 2008). In our study, WPI was used as a benchmark emulsifier to assess the impact of surface charge on bacterial growth, with pH variations accounted for by comparing emulsifier‐enriched environments to TSB at equivalent pH levels. Growth rate ratios under various emulsion and emulsifier conditions were statistically evaluated and presented in Table 3, revealing significantly accelerated growth rates in emulsions with negatively charged emulsifiers at equal oil concentrations (*p* < 0.05). Interestingly, at a 60% oil concentration, the cationic emulsifier (WPI^+^) exhibited a higher growth rate ratio than its anionic counterpart (*p* < 0.05), with a lag phase observed exclusively in WPI^+^‐stabilized 60% emulsions. These findings suggested a nuanced interaction between cationic emulsifiers, bacterial cells, and higher oil concentrations.

**TABLE 3:** Growth rate ratios for *S*. Typhimurium in WPI solutions and WPI‐stabilized emulsions (20%, 40%, and 60%) against TSB, as well as for WPI^+^ solutions and WPI^+^‐stabilized emulsions (20%, 40%, and 60%) relative to TSB adjusted to pH 5.0.

	Growth rate		Lag phase	
	TSB	TSB, pH = 5	TSB	TSB, pH = 5
WPI/WPI^+^	1.19 ± 0.09^a^	1.01 ± 0.02^b^	1.18 ± 0.22^A,B^	1.04 ± 0.07^A,B,C^
WPI/WPI^+^, 20% emulsion	1.18 ± 0.10^a^	0.84 ± 0.04^b,c^	1.11 ± 0.29^A,B,C^	0.94 ± 0.03^B,C,D^
WPI/WPI^+^, 40% emulsion	0.87 ± 0.08^b^	0.57 ± 0.03^d^	0.40 ± 0.35^D^	0.86 ± 0.07^C,D^
WPI/WPI^+^, 60% emulsion	0.37 ± 0.07^d^	0.78 ± 0.04^c^	NA	1.10 ± 0.01^A,B,C^

*Note:* The reported values represent the mean of three independent experiments, with the standard deviation (SD) provided to indicate the spread of the data. To discern significant variations in growth rates among the different groups, lowercase alphabetic labels (a, b, c, etc.) were applied, as determined by multiple *t*‐tests. Bonferroni correction was then proceeding to avoid Type I error. Similarly, uppercase letters (A, B) were used to highlight significant differences in lag phase durations among the groups, also derived from multiple *t*‐tests. Bonferroni correction was then proceeding to avoid Type I error. Groups assigned the same alphabetic label do not exhibit statistically significant differences (*p* > 0.05), whereas those marked with different letters do (*p* < 0.05).

### 3.3 Thermal Inactivation Dynamics of *S*. Typhimurium in Emulsions With Varied Emulsifier Characteristics

**FIGURE 2:**
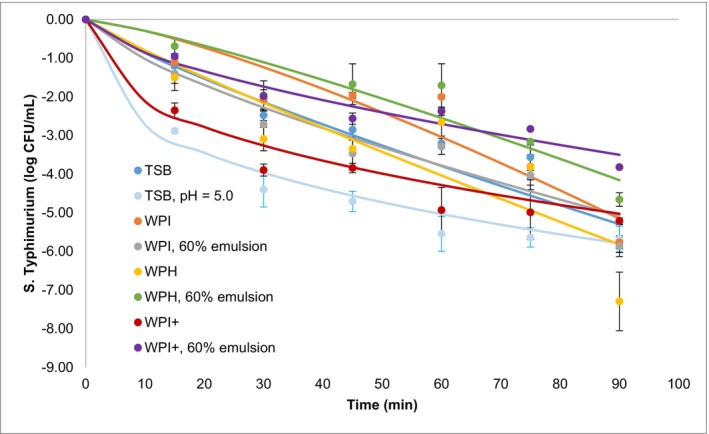
Thermal inactivation of *S*. Typhimurium at 55°C within various mediums: TSB, TSB adjusted to pH 5.0, and emulsions containing WPI, WPH, or WPI^+^ as emulsifiers, each with a 60% oil phase. The Weibull model was employed to fit the experimental observations, with solid lines illustrating the average fit across the inactivation curves. The values presented for each point represent the mean from three independent experiments, accompanied by the standard deviation (SD) to indicate variability.

The research aimed to explore the thermal inactivation behaviors of *S*. Typhimurium within emulsions, particularly focusing on 60% oil fraction emulsions and their influence on bacterial inactivation, an area previously touched upon by Tsai and Tikekar (2023). Figure 2 shows thermal inactivation trends for bacteria inoculated in TSB, emulsifier solutions and 60% (v/v) emulsions. Upon thermal treatment at 55°C, bacterial inactivation kinetics showed a nonlinear trend, with a notable tailing effect, especially with higher oil fractions in the emulsion, which showed the comparable result with Warren (1998) findings.

For the quantitative analysis of inactivation data, the Weibull model was applied. *β* value < 1 suggested bacterial adaptation to heat, whereas *β* value > 1 indicated a heightened sensitivity to thermal process. The *α* parameter, analogous to the traditional *D* value, was extended to calculate the 5*D* value, a measure of the efficacy of bacterial inactivation (Table 4). The analysis revealed no significant differences in the 5D value among WPI solution, WPI‐stabilized 60% emulsion, and WPH solution in comparison with TSB (*p* > 0.05). However, a significant difference was observed in the 5D value for the WPH‐stabilized 60% emulsion, which was significantly increased relative to TSB (*p* < 0.05), suggesting an enhanced resistance to thermal inactivation within this particular emulsion. In addition, while the 5*D* value for *S*. Typhimurium in WPI^+^ solution was higher when compared to TSB at a pH of 5.0 (*p* < 0.05), it was much higher in WPI^+^‐stabilized 60% emulsions (*p* < 0.05), implying additional protection offered by oil in cationic emulsifiers included environments under thermal stress. Such an observation aligns with the findings of Yang et al. (2020), who reported enhanced heat resistance in bacteria at lower water activity levels, achieved through higher oil content. Concurrently, Yegin et al. (2019) identified a potential for cationic emulsifiers to adhere to bacterial membranes. We hypothesize that this adherence could contribute to the observed protective effect during thermal treatment, potentially through a synergistic interaction between the oil content and cationic emulsifiers. The synergy between oil and cationic emulsifiers might bolster this protective effect on bacteria.

**TABLE 4:** Parameters derived from applying the Weibull model to thermal inactivation data of *S*. Typhimurium at 55°C in various mediums: TSB, TSB adjusted to pH 5.0%, and 60% emulsions stabilized with WPI, WPH, or WPI^+^.

	TSB	TSB, pH = 5.0	WPI	WPI, 60% emulsion	WPH	WPH, 60% emulsion	WPI^+^	WPI^+^, 60% emulsion
*α* (min)	4.33 ± 0.25^c^	0.05 ± 0.03^e^	13.41 ± 1.65^a^	3.09 ± 1.81^cd^	5.08 ± 0.67^c^	13.81 ± 2.80^a^	0.18 ± 0.17^e^	3.42 ± 0.99^c,d^
*β*	0.82 ± 0.02^c^	0.34 ± 0.03^e^	1.30 ± 0.09^a^	0.73 ± 0.13^c,d^	0.90 ± 0.03^b,c^	1.21 ± 0.11^a,b^	0.39 ± 0.07^e^	0.64 ± 0.06^d^
Avg. 5D predicted by Weibull model (min)	83.92 ± 2.33^c,d^	53.35 ± 7.83^e^	88.02 ± 1.88^b,c^	84.38 ± 2.28^c,d^	75.96 ± 8.12^d^	104.62 ± 3.60^b^	76.48 ± 11.96^d^	156.70 ± 13.28^a^

*Note:* The values listed represent the averages obtained from three replicate experiments, with the standard deviation (SD) provided to denote the variability. To identify significant differences in thermal inactivation parameters across the groups, alphabetic labels (a, b, c, etc.) were assigned based on the outcomes of multiple *t*‐tests. Bonferroni correction was then proceeding to avoid Type I error. Groups sharing a common letter do not show statistically significant differences (*p* > 0.05), whereas those marked with different letters exhibit significant disparities (*p* < 0.05).

The study further examined the influence of emulsifier surface charge on bacterial inactivation at 55°C by comparing WPI and WPI^+^ and their 60% oil emulsions, with the ratio between WPI included environments and TSB, and WPI^+^ included environments and TSB at pH = 5 (Table 5). Although no significant differences were found in the ratio of *α* and *β* values between emulsifiers with different charges (*p* > 0.05), a significant increase in these values was identified in 60% oil emulsion stabilized with WPI^+^ compared to those stabilized with WPI (*p* < 0.05). Moreover, higher 5*D* values were detected in environments containing WPI^+^ compared to those with WPI (*p* < 0.05). This difference in inactivation kinetics could be traced back to the surface charge of emulsifiers. Abbaszadegan et al. (2015) found that positively charged silver nanoparticles have a strong electrostatic attraction to negatively charged bacterial cells, enhancing their antibacterial efficacy. This suggests that positively charged emulsifiers, like those in WPI^+^ emulsion, may similarly interact with bacterial cells, potentially reducing their inactivation during thermal treatment. However, the protective effect on bacterial cells was not significant in the emulsifier solution alone; it was more pronounced when combined with oil.

**TABLE 5:** Comparative ratios of the Weibull model parameters α, β, and the 5D value for *S*. Typhimurium thermal inactivation in WPI solutions and WPI‐stabilized 60% emulsions relative to TSB, as well as for WPI^+^ solutions and WPI^+^‐stabilized 60% emulsions compared with TSB adjusted to pH 5.0.

	*α*	*β*	5*D* value
WPI	3.10 ± 0.38^b^	1.57 ± 0.11^b^	1.05 ± 0.02^c^
WPI, 60% emulsion	0.71 ± 0.42^b^	0.88 ± 0.15^c^	1.01 ± 0.03^c^
WPI^+^	3.66 ± 3.47^b^	1.14 ± 0.21^bc^	1.43 ± 0.22^b^
WPI^+^, 60% emulsion	70.28 ± 20.38^a^	1.85 ± 0.18^a^	2.94 ± 0.25^a^

*Note:* The data points reflect the mean values from three sets of measurements, with the standard deviation (SD) indicating the range of variation. Alphabetic labels (a, b, c, etc.) denote the significance levels of differences between the groups, as determined by multiple *t*‐tests. Bonferroni correction was then proceeding to avoid Type I error. Groups assigned the same letter do not exhibit statistically significant differences (*p* > 0.05), whereas distinct letters indicate a significant divergence in values (*p* < 0.05).

The study sheds light on the effect between emulsifier properties, oil fractions, and their collective impact on bacterial growth and inactivation. However, there are certain limitations that warrant acknowledgment. The choice of bacterial strain, *Salmonella* Typhimurium, while relevant, limits the scope to this particular microorganism. Additionally, the findings may exhibit variability with different emulsifiers and in diversified food matrixes. We also assume a consistent interaction between the emulsifiers and bacterial cells across all tested conditions, an aspect that might exhibit variability in real‐world food systems.

## 4 Conclusion

This study highlighted the significant influence of emulsifier characteristics, including Molecular weight (MW) and surface charge, as well as oil content, on the dynamics of *Salmonella* Typhimurium within oil‐in‐water emulsions. The employment of different emulsifiers, characterized by distinct MW and surface charges, had demonstrated some impacts on the behavior of *Salmonella* Typhimurium. The findings showed that lower MW emulsifiers, such as WPH, potentially moderated bacterial growth rates. In contrast, surface charge appeared to be a less influential factor on growth rates in emulsifier solutions, although its impact becomes pronounced in high oil fraction emulsions, particularly with cationic emulsifiers. The cationic emulsifier (WPI^+^) may offer a protective effect to bacteria against thermal inactivation as well. Additionally, the presence of oil was found to possibly shield bacteria from thermal treatment, especially in emulsions stabilized by lower MW or cationic emulsifiers. These insights emphasize a complex interaction between emulsifier properties, oil content, and bacterial growth and inactivation. Hence, selection of emulsifiers, considering both their MW and surface charge, alongside oil fraction levels, emerges as a vital consideration in formulating safer and higher quality emulsion‐based food products.

## Author Contributions

**Shawn Tsai:** conceptualization (supporting), data curation (lead), formal analysis (lead), investigation (lead), methodology (lead), software (lead), visualization (lead), writing – original draft (lead). **Rohan V. Tikekar:** conceptualization (equal), funding acquisition (lead), project administration (lead), resources (lead), supervision (lead), writing – review and editing (lead).

## Conflicts of Interest

The authors declare no conflicts of interest.

## Data Availability

Data are made available upon reasonable request by contacting the corresponding author.
